# Impact of inactivated vaccines on decrease of viral RNA levels in individuals with the SARS-CoV-2 Omicron (BA.2) variant: A retrospective cohort study in Shanghai, China

**DOI:** 10.3389/fpubh.2023.1107343

**Published:** 2023-03-07

**Authors:** Peng Yang, Bianli Dang, Wen Kang, Xiaofeng Li, Tianping Wang, Ruijuan Li, Meijuan Peng, Yushen Liu, Linxu Wang, Yan Cheng, Suhuai Yu, Min Wei, Han Gao, Wenzhen Kang, Lei Shang

**Affiliations:** ^1^Department of Health Statistics, Ministry of Education Key Lab of Hazard Assessment and Control in Special Operational Environment, School of Public Health, Fourth Military Medical University, Xi'an, China; ^2^Department of Infectious Diseases, The Second Affiliated Hospital, Fourth Military Medical University, Xi'an, China; ^3^The Third Regiment, Basic Medical Science Academy, Fourth Military Medical University, Xi'an, China

**Keywords:** COVID-19, SARS-CoV-2, Omicron, inactivated vaccines, viral RNA levels

## Abstract

**Background:**

SARS-CoV-2 Omicron (BA.2) has stronger infectivity and more vaccine breakthrough capability than previous variants. Few studies have examined the impact of inactivated vaccines on the decrease of viral RNA levels in individuals with the Omicron variant, based on individuals' continuous daily cycle threshold (Ct) values and associated medical information from the infection to hospital discharge on a large population.

**Methods:**

We extracted 39,811 individuals from 174,371 Omicron-infected individuals according to data inclusion and exclusion criteria. We performed the survival data analysis and Generalized Estimating Equation to calculate the adjusted relative risk (aRR) to assess the effect of inactivated vaccines on the decrease of viral RNA levels.

**Results:**

Negative conversion was achieved in 54.7 and 94.3% of all infected individuals after one and 2 weeks, respectively. aRRs were shown weak effects on turning negative associated with vaccinations in asymptomatic infections and a little effect in mild diseases. Vaccinations had a protective effect on persistent positivity over 2 and 3 weeks. aRRs, attributed to full and booster vaccinations, were both around 0.7 and had no statistical significance in asymptomatic infections, but were both around 0.6 with statistical significance in mild diseases, respectively. Trends of viral RNA levels among vaccination groups were not significant in asymptomatic infections, but were significant between unvaccinated group and three vaccination groups in mild diseases.

**Conclusion:**

Inactivated vaccines accelerate the decrease of viral RNA levels in asymptomatic and mild Omicron-infected individuals. Vaccinated individuals have lower viral RNA levels, faster negative conversion, and fewer persisting positive proportions than unvaccinated individuals. The effects are more evident and significant in mild diseases than in asymptomatic infections.

## Introduction

Coronavirus disease 2019 (COVID-19) remains a severe threat to global public health at present. The current primary variant is SARS-CoV-2 Omicron, which has unprecedented mutations, higher infectivity, and greater vaccine breakthrough capabilities than previous variants (1–3). Individuals with Omicron mostly present with asymptomatic or mild symptoms (4). Due to the lack of apparent signs of upper respiratory infection (such as cough, sputum, and sneezing) (3), infected individuals were usually delayed in isolation, which led to more secondary transmissions.

Vaccines have been shown to not only control the spread of disease but also prevent severe illness in infected individuals with the Alpha, Beta, and Delta strains of COVID-19 (5–8). Vaccines were slightly less protective against Omicron strains than previous strains (9). Two-dose vaccine provided limited and short-lived protection against the Omicron virus (10), and the booster shot was more effective than two and one-dose vaccines (11). The duration of antibody titer levels and protective effects against the Omicron virus varied depending on the type of vaccine (12). However, most previous studies on the impact of vaccination were focused on mRNA vaccines and were primarily based on clinical or laboratory data from relatively small samples (12–14). Although some articles analyzed the inactivated vaccines, their primary focus was on the safety and immunogenicity of vaccines (15–20). The article (21) examined the effect of inactivated vaccination on a large population in Hong Kong, but it did not analyze individuals' continuous cycle threshold (Ct) values from infection to hospital discharge.

A recent Omicron BA.2 outbreak caused a COVID-19 pandemic in Shanghai, one of China's most populated and economically developed metropolises, with almost 25 million inhabitants (22). In just 5 months from January 1 to May 31, 2022, despite 75% population vaccine coverage and a certain degree of non-pharmaceutical interventions, the total number of Omicron BA.2 infections reached 626,000 in Shanghai. According to local policies, all infected individuals with positive nucleic acid tests were centralized and quarantined timely at the Fangcang Shelter Hospital (FSH) of the National Exhibition and Convention Center (NECC). The FSH is the largest shelter hospital during the Omicron BA.2 pandemic and is equipped to support non-severe patients in Shanghai. Totally 174,371 asymptomatic infections and mild diseases were isolated in the FSH from April 9 to May 24, 2022. To monitor disease progression and promote patient recovery, each infected individual in the FSH was screened daily by the nucleic acid test with pharyngeal swab specimens until discharge after negative conversion.

In this study, we mainly performed the survival data analysis and Generalized Estimating Equation (GEE) to estimate the decrease of viral RNA levels among four vaccine groups in asymptomatic and mild Omicron infections, for the purpose of providing the calibration of future pandemic control measures based on inactivated vaccines in China.

## Materials and methods

### Data

All data were obtained from the FSH. Primary data included the daily Ct values from admission with confirmed positive diagnosis to discharge after turning negative. Data for each patient were obtained from the electronic medical record system, including individual age, gender, marital status, comorbidities, symptomatic status, admission date, discharge date, infection date, ORF1ab value, N gene value, and location of first positive screening. The length of stay was calculated by subtracting the admission date from the discharge date.

### Classification of vaccination status

Vaccination status was divided into four groups based on the vaccine doses received: unvaccinated, partially vaccinated, fully vaccinated, and booster. People who have received at least one vaccine dose but did not complete all doses prescribed by the vaccination protocol were defined as partially vaccinated. Those who had received all doses prescribed by the vaccination protocol were defined as fully vaccinated, while those who received a booster shot were regarded as the booster vaccination. Based on the immunization schedule, fully vaccinated was given 3 weeks after partial vaccinations, and booster vaccination was administered 6 months following full vaccinations.

According to Chinese epidemic prevention and control strategies (23) and Shanghai's requirement to promote free vaccination for the whole population (24, 25), vaccination time for residents in Shanghai was mostly concentrated. Individuals who administered partial vaccines were mainly focused between April and June 2021, the full vaccines were between May and July 2021, and boosters were between November 2021 and January 2022.

### Definitions of infection, turning negative, and persisting positive

The pharyngeal swab specimens were tested for the ORF1ab and N genes of SARS-CoV- 2 with real-time RT-PCR using the SARS-CoV-2 Detection Kit (Easy Diagnosis Biomedicine Co., Ltd., Wuhan, China) with MA6000 Real-Time PCR Detection System (Molarray Bioscience Co., Ltd., Suzhou, China) by Shanghai Labway Clinical Laboratory. A Ct value (ORF1ab or N genes) below 35 indicated a positive for SARS-CoV-2 RNA (26). For infected individuals during those periods in Shanghai, the majority of positive samples were confirmed as BA.2 sub-lineages (22, 27, 28); no other variants were identified.

According to the Diagnosis and Treatment Guideline for COVID-19 (Ninth Edition) (26) by the National Health Commission of China, an asymptomatic infection was defined as a patient who did not have any of the following symptoms: fever (>37.5°C), chills, myalgia, fatigue, rhinorrhea, nasal congestion, hyposmia, hypogeusia, sore throat, dyspnea, cough, sputum production, hemoptysis, headache, dizziness, anorexia, nausea, vomiting, abdominal pain, and diarrhea. The mild disease was defined as having any of the above symptoms, and additionally the symptoms are mild, the imaging examination showed no signs of pneumonia, and individuals had low oxygen saturation (≤ 93%).

Turning negative was defined as a decrease in viral RNA levels and Ct values were more than 35 by two consecutive negative nucleic acid tests with sampling intervals more than 24 h. Persisting positive was defined as viral RNA levels not decreasing and Ct values were always >35 from admission to this nucleic acid test. Infected individuals were eligible for discharge if they had a normal body temperature for more than 3 days and significant improvement in their symptoms in addition to turning negative.

### Data inclusion and exclusion criteria

We recruited all individuals with asymptomatic and mild symptoms of COVID-19. The first nucleic acid test after admission should be positive. Infected individuals should be isolated to the FSH on the day or the next day when they were confirmed positive (no more than 2 days between positive diagnosis and admission). Infected individuals with a Ct value of more than 35 on the day of admission, a hospital stay of fewer than 3 days, or no continuous nucleic acids after admission were all excluded. Individuals vaccinated with non-inactivated vaccines (e.g., adenovirus type 5 vector vaccines, recombinant protein subunit vaccines) were not included in the study. Missing values and outliers were omitted. The flow chart of study design was shown in Supplementary Figure 1.

### Study design and statistical method

Since individuals in the FSH only received oral medications for their complications and were devoid of antiviral and immune-boosting drugs (which can affect nucleic acid conversion), it is reasonable to observe the impact of inactivated vaccines on the decrease of viral RNA levels in Omicron-infected individuals.

We treated the occurrence of individual's negative conversion as the binary dependent variable and used the length of stay as the survival time. We then used multivariate Cox regression, adjusted by other influential variables, to analyze adjusted relative risk (aRR) for the effect of four types of vaccinations on the decrease of viral RNA levels, respectively. We then treated whether the patient was persisting positive (1 indicates yes, 0 indicates not) at the 2- and 3-week as time points, respectively, adjusted by influential variables, to analyze the aRR for persisting positive associated with vaccinations through multivariable logistic regression.

We analyzed the trends of viral RNA decay among four vaccination groups through the GEE. In the model, continuous Ct values for O and N genes were considered as dependent variables, respectively. Type of vaccination was as the factor variable, influential variables (such as age, gender, hypertension, diabetes, marital status) as covariates, and id (a vector which identifies the clusters of individual repeated measurement) as the subject variable. The paired comparisons between groups were based on Fisher's Permutation test.

The *Chi*-square test was used to compare categorical variables. *Kruskal-Wallis H* test was used to compare the differences in age (years), duration (days), and Nadir Ct values among vaccination groups of unvaccinated, partial, full, and booster. All the analyses were performed in *R* software (version 4.1.2). The Cox regression was constructed with the “survival” and “survminer” packages, GEE with “geepack” package, Fisher's Permutation test with “EnvStats” package. The logistic regression was calculated using the “glm” function, *P* < 0.05 indicates a statistical significance.

## Results

### Characteristics of individuals with Omicron infection by vaccination type

**Figure 1:**
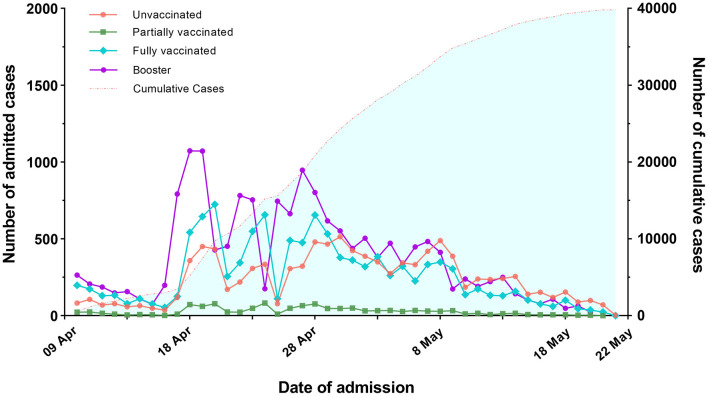
Trends in the number of individuals admitted to the FSH among four types of vaccinations.

A total of 39,811 individuals were enrolled in the study. 70.6% of them received full or booster vaccination, 3.0% were partially vaccinated, and 26.4% were unvaccinated (Table 1). Figure 1 depicts the trends in the number of infected individuals admitted to the FSH among four types of vaccination from April 9, 2022 to May 22, 2022.

**Table 1:** Characteristics of individuals infected with Omicron classified by type of vaccination.

**Characteristics**	**Total**	**Classification of vaccination**				***P* value**
		**Unvaccinated**	**Partially vaccinated**	**Fully vaccinated**	**Booster**	
**No. of cases (%)**	**39,811**	**10,515 (26.4%)**	**1,202 (3.0%)**	**11,504 (28.9%)**	**16,590 (41.7%)**	
**Age, years, Median (IQR** ^a^ **)**	42 (31–55)	46 (32–59)	33 (26–45)	36 (26–52)	46 (33–55)	<0.001^b^
≤ 18	1,662 (4.2)	593 (5.6)	70 (5.8)	967 (8.4)	32 (0.2)	<0.001^c^
18–30	8,281 (20.8)	1,697 (16.1)	434 (36.1)	3,073 (26.7)	3,077 (18.5)	
31–40	8,791 (22.0)	2,081 (19.8)	330 (27.5)	2,754 (23.9)	3,626 (21.9)	
41–50	6,962 (17.5)	1,671 (15.9)	155 (12.9)	1,598 (13.9)	3,538 (21.3)	
51–60	8,678 (21.8)	2,180 (20.7)	127 (10.6)	1,840 (16.0)	4,531 (27.3)	
61–70	4,842 (12.2)	1,979 (18.8)	76 (6.3)	1,146 (10.0)	1,641 (9.9)	
≥71	595 (1.5)	314 (3.0)	10 (0.8)	126 (1.1)	145 (0.9)	
**Gender**, ***n*** **(%)**						<0.001^c^
Female	16,339 (41.0)	4,341 (41.3)	386 (32.1)	4,830 (42.0)	6,782 (40.9)	
Male	23,472 (59.0)	6,174 (58.7)	816 (67.9)	6,674 (58.0)	9,808 (59.1)	
**Marital status**, ***n*** **(%)**						<0.001^c^
Married	23,244 (58.4)	5,808 (55.2)	499 (41.5)	6,012 (52.3)	10,925 (65.9)	
Unmarried	15,163 (38.1)	4,268 (40.6)	654 (54.4)	5,133 (44.6)	5,108 (30.8)	
Others	1,404 (3.5)	439 (4.2)	49 (4.1)	359 (3.1)	557 (3.4)	
**Comorbidities**, ***n*** **(%)**						
Hypertension	4,624 (8.4)	845 (8.0)	58 (4.8)	817 (7.1)	1,357 (8.2)	<0.001^c^
Diabetes	1,633 (3.0)	405 (3.9)	26 (2.2)	294 (2.6)	385 (2.3)	<0.001^c^
**Length of stay, days, Median (IQR)**	8 (7–11)	9 (7–11)	8 (7–10)	8 (7–10)	8 (7–10)	<0.001^b^
**Time to negative conversion, days, Median (IQR)**	6 (5–8)	7 (5–9)	6 (5–8)	6 (5–8)	6 (5–8)	<0.001^b^
**Nadir Ct values, Median (IQR)**						
ORF1ab gene	28.8 (25.7–32.0)	28.3 (25.2–31.5)	28.9 (26.0–32.2)	29.0 (25.8–32.1)	29.0 (25.9–32.1)	<0.001^b^
N gene	27.1 (24.0–30.1)	26.6 (23.5–29.6)	27.2 (24.2–30.2)	27.2 (24.1–30.2)	27.2 (24.3–30.2)	<0.001^b^
**Location of first positive screening**, ***n*** **(%)**						<0.001^c^
Community screening	15,543 (39.0)	3,281 (31.2)	489 (40.7)	5,214 (45.3)	6,659 (39.5)	
Companies/schools screening	5,870 (14.7)	593 (5.6)	175 (14.6)	1,762 (15.3)	3,340 (20.1)	
Active screening at nucleic acid sampling points	5,847 (14.7)	941 (8.9)	217 (18.1)	1,965 (17.1)	2,724 (16.4)	
Fever clinic screening	12,551 (31.5)	5,700 (54.2)	321 (26.7)	2,563 (22.3)	3,967 (23.9)	
**Symptomatic status**, ***n*** **(%)**						<0.001^c^
Asymptomatic infection	6,809 (17.1)	2,024 (19.2)	214 (17.8)	1,901 (16.5)	2,670 (16.1)	
Mild disease	33,002 (82.9)	8,491 (80.8)	988 (82.2)	9,603 (83.5)	13,920 (83.9)	

^a^IQR, interquartile range (P_25_-P_75_). ^b^P-value calculated by Kruskal–Wallis test. ^c^P-value calculated by Chi-squared test.

Of all individuals, 17.1% were asymptomatic infections and 82.9% were mild diseases. 41.0% of the total were females and 59.0% were males. The average age was 42 years [Interquartile Range(IQR): 31–55]. There were 1,662 (4.2%) individuals aged ≤ 18 years, 32,712 (82.1%) aged 19 to 60 years, and 5,437 (13.7%) aged ≥60 years; 8.4 and 3.0% of all individuals had hypertension and diabetes, respectively.

The average length of hospital stay was 8 days [IQR: 7–11] and median time to negative conversion was 6 days [IQR: 5–8]. The ORF1ab gene averaged 28.8 [IQR: 25.7–32.0], slightly higher than the N gene average of 27.1 [IQR: 24.0–30.1]. Among locations of first positive screening, community screening had the highest proportion at 39.0%, followed by the fever clinic screening at 31.5%.

### Relative risk for negative conversion associated with vaccinations

**Figure 2:**
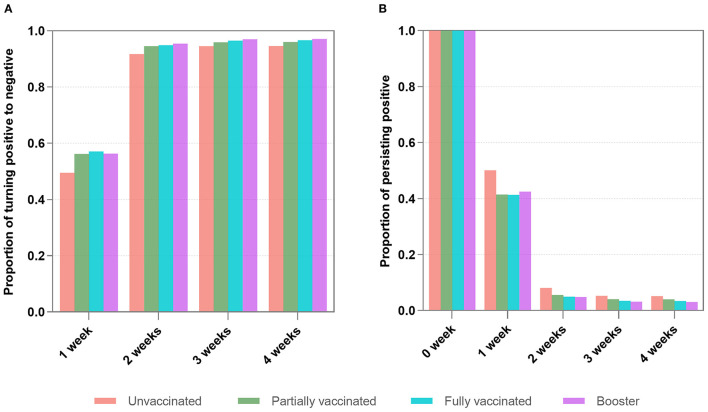
The proportions of turning positive to negative **(A)** and persisting positive **(B)** within 4 weeks. **(A)** Shows that 54.7% of infected individuals achieved negative conversion after 1 week, and 94.3% achieved negative conversion after 2 weeks (2 weeks *vs. 1 week, P* < 0.001). There were significant differences in negative changes between 3-, 4, and 1-week (*P* < 0.001), but they were not statistically significant between the three and 4 weeks (*P* = 0.48). **(B)** Reveals that 6.5% of infected individuals remained positive after 2 weeks. There were no significant differences between the proportions after three and 4 weeks (*P* = 0.32).

Figure 2A shows that 54.7% of infected individuals turned positive to negative after 1 week, and 94.3% turned positive to negative after 2 weeks (2 *vs. 1 week, P* < 0.001). 96.2% turned positive to negative after 3 weeks, and 96.4% turned positive to negative after 4 weeks. The change in the proportion of negative conversion become slow after 2 weeks. There were significant differences in negative conversion between 3-, 4-, and 1-week (*P* < 0.001), but they were not statistically significant between the 3 and 4 weeks (*P* = 0.48).

**Figure 3:**
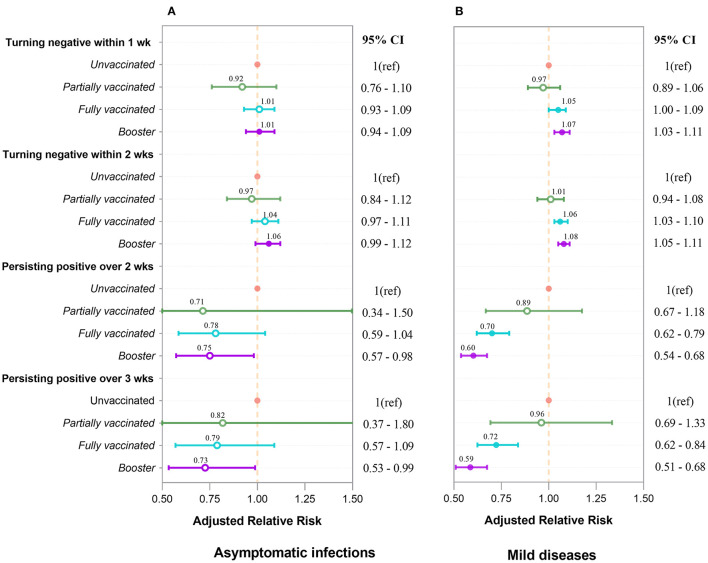
Adjusted relative risk for turning negative and persisting positive associated with four types of vaccination in asymptomatic infections **(A)** and mild diseases **(B)**. aRRs were analyzed by Cox regression and logistic regression. Inactivated vaccines accelerate negative conversion and prevent persisting positive. The booster's effect outperforms that of full vaccination, which outperforms that of partial vaccination. These effects are more pronounced and significant in mild diseases **(B)** than in asymptomatic infections **(A)**. Each point and interval in the figure represent the aRR and 95% confidence interval. Circles for points are filled if the value is significant (*P* < 0.05), and open otherwise.

**Figure 4:**
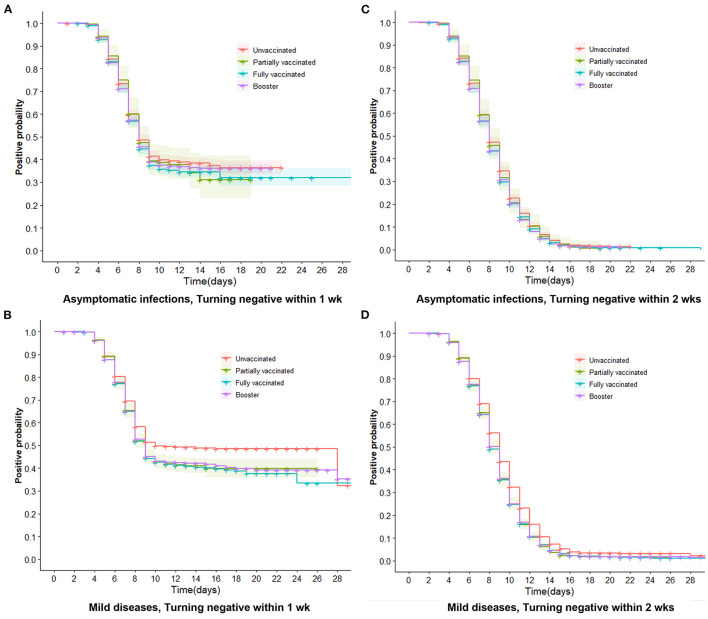
Trends of negative conversion within 1 week and 2 weeks **in** asymptomatic infections and mild diseases. Trends of negative conversion among four vaccine groups were analyzed by the Cox regression. Curves of four types of vaccinations are similar and close together for turning negative within 1 week and 2 weeks in asymptomatic infections **(A, C)**. The booster, full, and partial vaccination curves are lower than the unvaccinated curve **(B, D)**, indicating that vaccinated individuals have a faster decline in viral RNA levels than unvaccinated individuals (*P* < 0.001).

We further performed multivariate Cox regression, classified by symptomatic status and adjusted by age, gender, marital status, comorbidities, nadir Ct value, and location of first positive screening, to observe the aRR of turning positive to negative associated with vaccinations. Figure 3A indicates that in asymptomatic infections, whether turning negative within 1 or 2 weeks, aRRs related to partial and full vaccination were mainly around one and had no significance. aRRs associated with booster were slightly bigger than one and statistically significant (*P* < 0.001). Moreover, we discovered that curves of the three vaccination groups were similar and close together for negative conversion within 1 and 2 weeks (Figures 4A, C).

For mild diseases (Figure 3B), vaccinated individuals were more likely to achieve negative conversion than unvaccinated individuals, regardless of whether negative conversion occurred within 1 or 2 weeks. The aRRs of negative conversion within 1 week were 1.05 (95% CI 1.00–1.09) and 1.07 (95% CI 1.03–1.11) in fully vaccinated and booster-vaccinated individuals, respectively. Within 2 weeks, the aRRs were 1.06 (95% CI 1.03–1.10) and 1.08 (95% CI 1.05–1.11). Figures 4B, D reveal that curves of booster, full, and partial vaccination are lower than the unvaccinated curve.

### Relative risk for persistent positivity associated with vaccinations

Figure 2B reveals that among the infected individuals with persisting positive, 6.5% of them remained positive after 2 weeks. After 3 and 4 weeks, the proportions continued to decrease slowly. There were no significant differences between the proportions after 3 and 4 weeks (*P* = 0.32).

By treating whether the patient was persisting positive as the binary dependent variable, we used the multivariate logistic analysis, adjusted by age, gender, marital status, comorbidities, nadir Ct values, and locations of the first swab for diagnosis, to investigate the aRR of persistent positivity attributed to types of vaccinations grouped by symptomatic status. Figure 3A depicts that vaccination had a protective effect on persistent positivity over 2 and 3 weeks in asymptomatic infections. The aRRs for partial, full, and booster vaccinations were all less than one but not statistically different.

Figure 3B shows that vaccinated individuals are less likely to have persistent positivity than unvaccinated individuals with mild disease, regardless of whether the persistent positivity occurred over 2 or 3 weeks. Furthermore, fully vaccinated individuals are less likely to have persistent positivity than partially vaccinated individuals, and booster individuals are less likely than fully vaccinated individuals to develop persistent positivity. For full and booster vaccination, aRRs for persistent positivity over 2-week were 0.70 (95% CI 0.62–0.79) and 0.60 (95% CI 0.54–0.68), respectively (*P* < 0.001). aRRs for persistent positivity over 3-week were 0.72 (95% CI 0.62–0.84) and 0.59 (95% CI 0.51–0.68), respectively (*P* < 0.001).

### Trends of viral RNA decay among four vaccination groups

Supplementary Table 1 shows that three types of vaccination all help to promote the decrease of viral RNA for both ORF1ab gene and N gene. Compared to unvaccinated, booster was the most effective, followed by fully and partially vaccinated. aRR values for the mild disease were generally higher than aRR values for asymptomatic patients. In asymptomatic infections, the aRRs for O genes were 1.24 (95% CI 1.14–1.34), 1.21 (95% CI 1.10–1.33) and 1.03 (95% CI 0.85–1.26). The aRRs for N genes were 1.26 (95% CI 1.21–1.31), 1.22 (95% CI 1.18–1.28) and 1.20 (95% CI 1.10–1.32). In mild diseases, the aRR for O genes were 1.29 (95% CI 1.18–1.42), 1.27 (95% CI 1.14–1.41), and 1.05 (95% CI 0.84–1.30). aRR of N genes 1.33 (95% CI 1.28–1.39), 1.28 (95% CI 1.22–1.34), and 1.27 (95% CI 1.14–1.40), respectively.

**Figure 5:**
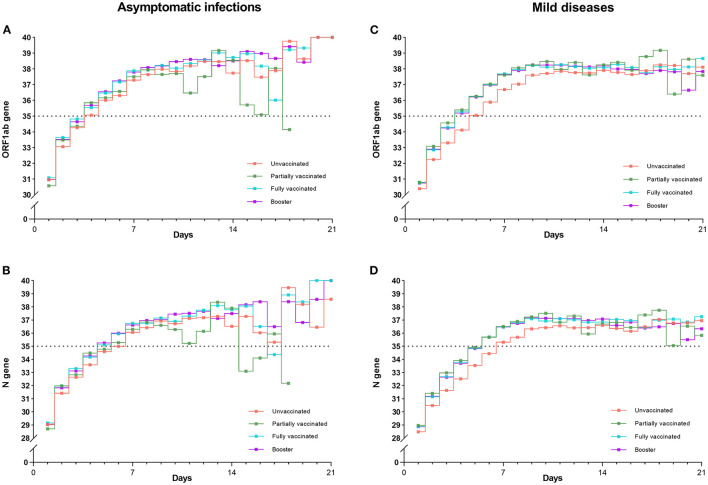
Trends of mean Ct values (ORF1ab and N genes) for four vaccinations in asymptomatic infections and mild diseases. Trends of Ct values for four vaccinations were almost the same in asymptomatic infections **(A, B)**. In contrast, the Ct values of the unvaccinated individuals were significantly lower than those of the other three vaccination groups in mild diseases **(C, D)** (*P* < 0.001).

Figures 5A, B show that, as viral RNA levels decreased, trends of Ct values (ORF1ab gene and N gene) of four vaccinations were almost the same in asymptomatic infections. In contrast, Figures 5C, D illustrate that in mild diseases, the Ct values of unvaccinated individuals were significantly lower than those of the other three vaccination groups (*P* < 0.001), while Ct values did not differ significantly between the three vaccination groups. *P* values of paired comparisons for ORF1ab gene and N gene were all more than 0.05.

## Discussion

As far as we know, few studies examined the effect of inactivated vaccines on viral RNA levels using continuous Ct values on a large population. In this study, we extracted continuous daily Ct values and associated medical risk information of 39,811 individuals from 174,371 Omicron-infected individuals, and conducted a comprehensive analysis to explore the impact of inactivated vaccines on the decrease of viral RNA levels. We found that inactivated vaccines could facilitate the decrease of viral RNA levels in infected individuals to some extent. Vaccinated individuals had lower viral RNA levels and turned negative faster than unvaccinated individuals in mild diseases. Booster vaccination outperformed full and partial vaccination. The effects are more evident and significant in mild diseases than in asymptomatic infections.

Vaccination can stimulate humoral immunity and cellular immune response. Neutralizing antibodies induced by vaccination might bind to the viral particles rendering those particles non-infectious. Although the vaccine does not prevent infection, it makes the immune system respond more rapidly and effectively when exposed to the pathogen again, helping to decrease the viral RNA levels and clear the virus (29). Individuals with mild diseases may be more likely to produce neutralizing antibody responses that facilitate decreasing viral RNA levels, making vaccination more effective in mild diseases than in asymptomatic infections, which is consistent with a previous report that neutralizing antibody has a high correlation with COVID-19 severity (30). mRNA vaccines were reported to be less effective in controlling and eliminating the Omicron virus than delta virus. Booster vaccine outperformed the two-dose and one-dose vaccines (13, 14, 21, 31–33). Our findings on the efficacy of the inactivated vaccine are consistent with previous research.

The population in Shanghai was mainly vaccinated with domestically-produced inactivated vaccine, which had relatively lower effectiveness in preventing SARS-CoV-2 infections due to their lower antibody-neutralizing responses compared to mRNA vaccines (21, 34). However, we found that, although the inactivated vaccine had a weak effect on turning negative (aRR slightly larger than 1), it had a better effect on preventing the continuous positive. The booster had the lowest aRR of 0.59 (95% *CI*: 0.51–0.68).

As of April 18, 2022, 91.4% of the population in Shanghai were vaccinated full primary schedule of COVID-19 vaccine program, and 53.7% of them received a booster (35). We found that the full primary schedule in the FSH of NECC was 71.9% and the booster shot was only 42.7%, which were significantly lower (*P* < 0.001) than the total vaccination coverage rate in Shanghai. The fact that infected population usually had a lower vaccination rate and uninfected population had a higher vaccination rate may further suggest that vaccination could reduce the risk of Omicron infection.

The viral RNA level reflects the severity of individuals' infections. We found that Omicron-infected individuals were shown a consistent and rapid decay in viral RNA levels over time among four vaccination groups. Almost all of individuals' Ct values had exceeded 35 by about 1 week. The results are in line with previous researches (36, 37). We further discovered that trend lines of unvaccinated individuals were lower than those of vaccinated individuals for both ORF1ab gene and N gene (Figure 5). This tendency was still observed after 2 weeks: although four lines all went negative, the lines of unvaccinated individuals were slightly lower than those of the vaccinated individuals. These results further confirmed that vaccination decreased the viral RNA levels in individuals with Omicron infections (38).

In addition, we made a further analysis of the elderly (>60) and found that (Supplementary Table 2), compared to the whole population, the proportion of unvaccinated elderly people is higher (42.2%), which may be related to that some older people are inconvenient to move and cannot go to the vaccination site for vaccination. The proportions of older people with hypertension (24.4%) and diabetes (9.5%) were also higher. Meanwhile, the days of length of stay and days of negative conversion for older people were 9 (7–12) and 7 (5–9), respectively, which were higher than the 8 (7–11) and 6 (5–8) for the whole population. Nadir Ct values of ORF1ab gene and N gene were 27.4 (24.5–30.6) and 25.6 (22.8–28.8), lower than the whole population of 28.8 (25.7–32.0) and 25.6 (22.8–28.8), respectively. These may be associated with a lower immunity in the elderly than in other age groups, resulting in a higher viral load after infection, leading to longer days in hospital and days to negative conversion. Furthermore, we found that the effects of types of vaccines were largely consistent between the elderly and whole population. The effect was more pronounced in mild (*P* < 0.001) than in asymptomatic (ORF1ab gene, *P* = 0.16; N gene, *P* = 0.06), and the booster was more effective than the Fully vaccinated and unvaccinated.

Compared to other studies, this paper has some advantages. First, to control the epidemic as much as possible, Shanghai conducted continuous nucleic acid tests for the whole population. Once a positive case was screened, isolation was implemented immediately. Therefore, data on infections who were admitted to the FSH within 2 days can be obtained. Second, Ct levels, symptom information, and associated influential factors were collected for continuous daily monitoring after admission. Third, infected individuals in the FSH only received oral medications for their complications and were mostly without antiviral and immune-boosting drugs, so it is reasonable to observe the impact of inactivated vaccines on viral RNA levels in Omicron-infected individuals. Finally, to our knowledge, the number of Omicron infections in our study is the largest report to date.

Some limitations must be acknowledged. Firstly, although the FSH is the largest shelter hospital in Shanghai, these data are only from the FSH and not from all infected individuals in Shanghai, which may lead to selection bias and affect the interpretation of results. Secondly, all individuals admitted in this study were asymptomatic infections and mild diseases, and there were no infected individuals with severe infections. Thirdly, we lack the data on the duration time of latest vaccination to admission. Although we can estimate that the duration time after vaccination is approximately the same within different vaccination groups based on Shanghai's immunization strategies, this would affect the interpretation of results to some extent when comparing vaccine effects between different groups. Lastly, no CT images of the patient's chest or other laboratory tests are not included in the data.

## Conclusions

Inactivated vaccinations accelerate the decrease of viral RNA levels in Omicron-infected individuals. Compared to unvaccinated individuals, vaccinated individuals have a lower viral RNA level, a faster negative conversion, and a smaller proportion of persisting positives. The impact of inactivated vaccines is more obvious and significant in mild diseases than in asymptomatic infections. This paper could provide the calibration of future pandemic control strategies based on inactivated vaccines in China.

## Data availability statement

The raw data supporting the conclusions of this article will be made available by the authors, without undue reservation.

## Ethics statement

The studies involving human participants were reviewed and approved by Clinical Research and Ethics Committee of Tangdu Hospital of the Fourth Military Medical University (No. 202205-01). The patients/participants provided their written informed consent to participate in this study.

## Author contributions

LS and WenzK conceived, designed, and supervised the study. PY, BD, and WenK co-prepared the first draft of the manuscript. PY analyzed the data and prepared the figures. XL, TW, RL, MP, YL, LW, YC, SY, MW, and HG provided critical revision of the manuscript. All authors read and approved the final manuscript.
