# Supplementary figures and images for: Impact of inactivated vaccines on decrease of viral RNA levels in individuals with the SARS-CoV-2 Omicron (BA.2) variant: A retrospective cohort study in Shanghai, China
Source: Front Public Health. 2023 Mar 7;11:1107343. doi: 10.3389/fpubh.2023.1107343 (PMC10028203; doi:10.3389/fpubh.2023.1107343)

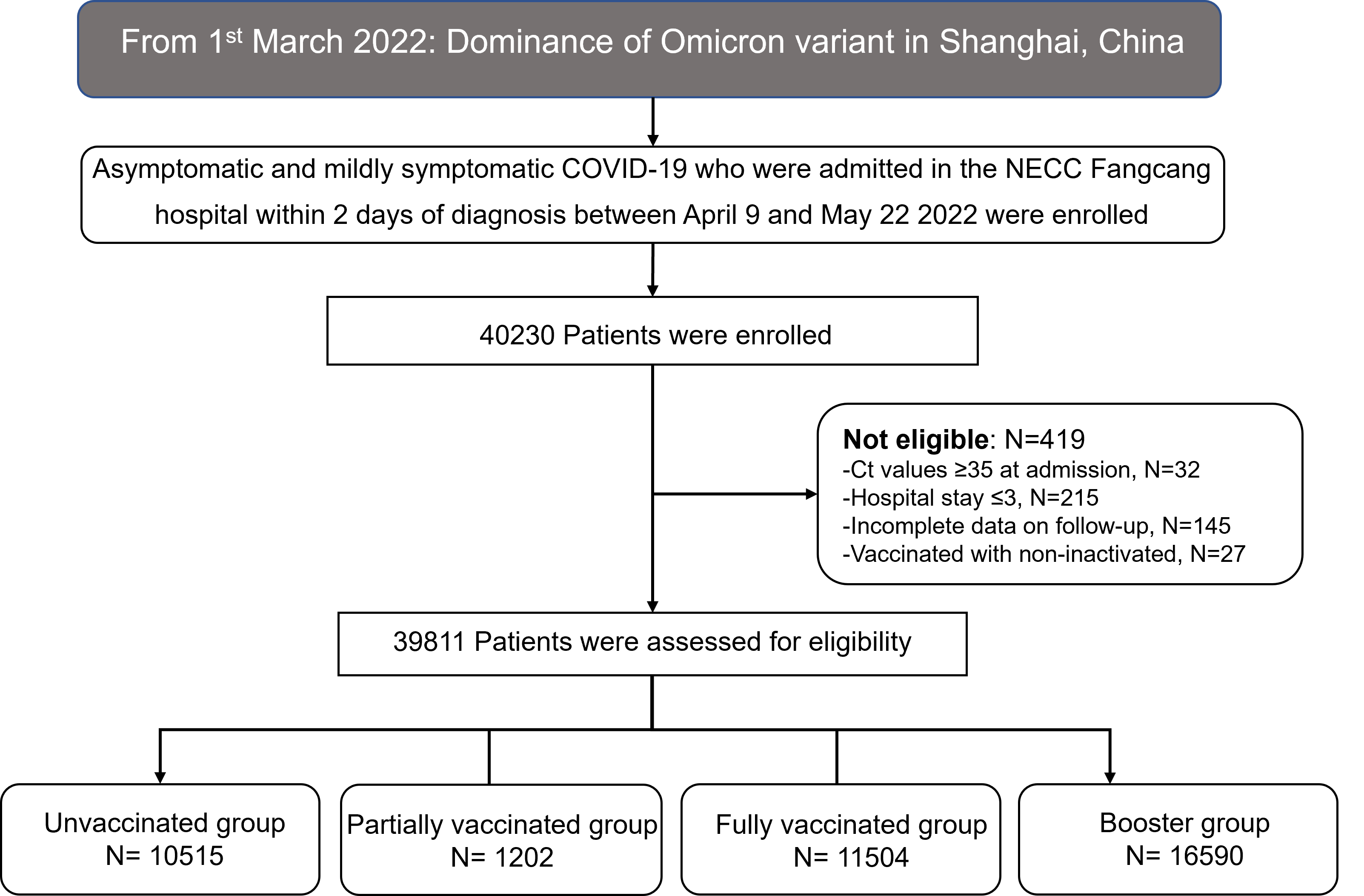

Supplement: Supplementary file 1 [file Image_1.tif]
