# Supplementary material for: Impact of inactivated vaccines on decrease of viral RNA levels in individuals with the SARS-CoV-2 Omicron (BA.2) variant: A retrospective cohort study in Shanghai, China
Source: Front Public Health. 2023 Mar 7;11:1107343. doi: 10.3389/fpubh.2023.1107343 (PMC10028203; doi:10.3389/fpubh.2023.1107343)
Supplement: Supplementary file 2 [file Data_Sheet_1.docx]

Supplementary Tables

**Table S1. Relative risk of viral RNA decay among four vaccination groups**

| **Vaccination** | **Asymptomatic** **infections** | |  | **Mild diseases** | |
| --- | --- | --- | --- | --- | --- |
|  | RR (95% CI) | *P* value |  | RR (95% CI) | *P* value |
| ORF1ab gene |  |  |  |  |  |
| Unvaccinated | 1 |  |  | 1 |  |
| Partially vaccinated^1^ | 1.03(0.85-1.26) | 0.75 |  | 1.05(0.84-1.30) | 0.70 |
| Fully vaccinated^2^ | 1.21(1.10-1.33) | <0.001 |  | 1.27(1.14-1.41) | <0.001 |
| Booster^3^ | 1.24(1.14-1.34) | <0.001 |  | 1.29(1.18-1.42) | <0.001 |
| N gene |  |  |  |  |  |
| Unvaccinated | 1 |  |  | 1 |  |
| Partially vaccinated^1^ | 1.20(1.10-1.32) | <0.001 |  | 1.27(1.14-1.40) | <0.001 |
| Fully vaccinated^2^ | 1.22(1.18-1.28) | <0.001 |  | 1.28(1.22-1.34) | <0.001 |
| Booster^3^ | 1.26(1.21-1.31) | <0.001 |  | 1.33(1.28-1.39) | <0.001 |

^1^Partially vaccinated *vs* Unvaccinated, ^2^Fully vaccinated *vs* Unvaccinated, ^3^Booster *vs* Unvaccinated

**Table S2. Characteristics of elderly (>60) infected with Omicron classified by type of vaccination**

| Characteristics | Total | Classification of vaccination | | | | *P* value |
| --- | --- | --- | --- | --- | --- | --- |
|  |  | Unvaccinated | Partially vaccinated | Fully vaccinated | Booster |  |
| No. of cases (%) | 5437 | 2293  (42.2%) | 86  (1.6%) | 1272  (23.4%) | 1786  (32.8%) |  |
| Age, years, Median (IQR^1^) | 66(63-68) | 66(64-69) | 65(64-68) | 65(63-68) | 65(63-68) | <0.001^2^ |
| 60~65 | 2678(49.3) | 1063(46.4) | 45(52.3) | 650(51.1) | 920(51.5) | <0.001^3^ |
| 65-70 | 2164(39.8) | 916(39.9) | 31(36.0) | 496(39.0) | 721(40.4) |  |
| ≥71 | 595(10.9) | 314(13.7) | 10(11.7) | 126(9.9) | 145(8.1) |  |
| Gender, n (%) |  |  |  |  |  | 0.22^3^ |
| Female | 2523(46.4) | 1073(46.8) | 34(39.5) | 611(48.0) | 805(45.1) |  |
| Male | 2914(53.6) | 1220(53.2) | 52(60.5) | 661(52.0) | 981(54.9) |  |
| Marital status, n (%) |  |  |  |  |  | <0.001^3^ |
| Married | 4201(77.3) | 1654(72.1) | 65(75.6) | 1022(80.3) | 1460(81.7) |  |
| Unmarried | 928(17.1) | 484(21.1) | 17(19.8) | 188(14.8) | 239(13.4) |  |
| Others | 308(5.7) | 155(6.8) | 4(4.7) | 62(4.9) | 87(4.9) |  |
| Comorbidities, n (%) |  |  |  |  |  |  |
| Hypertension | 1326(24.4) | 514(22.4) | 12(14.0) | 336(26.4) | 464(26.0) | <0.002^3^ |
| Diabetes | 518(9.5) | 240(10.5) | 6(7.0) | 125(9.8) | 147(8.2) | <0.09^3^ |
| Length of stay, days, Median (IQR) | 9(7-12) | 10(8-12) | 9(7-12) | 9(7-12) | 9(7-11) | <0.001^3^ |
| Time to negative conversion, days, Median (IQR) | 7(5-9) | 8(5-10) | 7(5-9) | 7(5-9) | 7(5-9) | <0.001^3^ |
| Nadir Ct values,  Median (IQR) |  |  |  |  |  |  |
| ORF1ab gene | 27.4  (24.5-30.6) | 27.1  (24.2-30.4) | 27.7  (24.9-30.5) | 27.5  (24.5-30.6) | 27.7  (24.8-30.9) | <0.003^2^ |
| N gene | 25.6  (22.8-28.8) | 25.4  (22.5-28.6) | 26.1  (23.4-28.5) | 25.7  (22.7-28.7) | 25.9  (23.1-29.1) | <0.003^2^ |
| Location of first  positive screening, n (%) |  |  |  |  |  | <0.001^3^ |
| Community screening | 2743(50.5) | 988(43.1) | 42(48.8) | 726(57.1) | 987(55.3) |  |
| Companies/schools screening | 207(3.8) | 52(2.3) | 6(7.0) | 55(4.3) | 94(5.3) |  |
| Active screening at nucleic acid sampling points | 532(9.8) | 172(7.5) | 12(14.0) | 117(9.2) | 233(13.0) |  |
| Fever clinic screening | 1953(35.9) | 1081(47.1) | 26(30.2) | 374(29.4) | 472(26.4) |  |
| Symptomatic status, n (%) |  |  |  |  |  | 0.16^3^ |
| Asymptomatic infection | 1047(19.3) | 463(20.2) | 18(20.9) | 253(19.9) | 313(17.5) |  |
| Mild disease | 4390(80.7) | 1830(79.8) | 68(79.1) | 1019(80.1) | 1473(82.5) |  |

^1^ IQR: interquartile range (P_25_-P_75_). ^2^ *P*-value calculated by *Kruskal–Wallis* test. ^3^ *P*-value calculated by *Chi-*squared test.
